# The Complete Chloroplast Genomes of *Echinacanthus* Species (Acanthaceae): Phylogenetic Relationships, Adaptive Evolution, and Screening of Molecular Markers

**DOI:** 10.3389/fpls.2018.01989

**Published:** 2019-01-10

**Authors:** Chunming Gao, Yunfei Deng, Jun Wang

**Affiliations:** ^1^College of Biological and Environmental Engineering, Binzhou University, Binzhou, China; ^2^Shandong Provincial Key Laboratory of Eco-Environmental Science for the Yellow River Delta, Binzhou University, Binzhou, China; ^3^Shandong Provincial Engineering and Technology Research Center for Wild Plant Resources Development and Application of Yellow River Delta, College of Biological and Environmental Engineering, Binzhou University, Binzhou, China; ^4^Key Laboratory of Plant Resources Conservation and Sustainable Utilization, South China Botanical Garden, Chinese Academy of Sciences, Guangzhou, China; ^5^Southeast Asia Biodiversity Research Institute, Chinese Academy of Sciences, Yezin, Myanmar

**Keywords:** *Echinacanthus*, chloroplast genome, sequence divergence, phylogeny, molecular marker, adaptive evolution

## Abstract

Among the four species of *Echinacanthus* (Acanthaceae), one distributed in the West Himalayan region and three restricted to the Sino-Vietnamese karst region. Because of its ecological significance, molecular markers are necessary for proper assessment of its genetic diversity and phylogenetic relationships. Herein, the complete chloroplast genomes of four *Echinacanthus* species were determined for the first time. The results indicated that all the chloroplast genomes were mapped as a circular structure and each genomes included 113 unique genes, of which 80 were protein-coding, 29 were tRNAs, and 4 were rRNAs. However, the four cp genomes ranged from 151,333 to 152,672 bp in length. Comparison of the four cp genomes showed that the divergence level was greater between geographic groups. We also analyzed IR expansion or contraction in the four cp genomes and the fifth type of the large single copy/inverted repeat region in Lamiales was suggested. Furthermore, based on the analyses of comparison and nucleotide variability, six most divergent sequences (*rrn16*, *ycf1*, *ndhA*, *rps16*-*trnQ*-UUG, *trnS*-GCU-*trnG*-UCC, and *psaA*-*ycf3*) were identified. A total of 37–45 simple sequence repeats were discovered in the four species and 22 SSRs were identified as candidate effective molecular markers for detecting interspecies polymorphisms. These SSRs and hotspot regions could be used as potential molecular markers for future study. Phylogenetic analysis based on Bayesian and parsimony methods did not support the monophyly of *Echinacanthus*. The phylogenetic relationships among the four species were clearly resolved and the results supported the recognition of the Sino-Vietnamese *Echinacanthus* species as a new genus. Based on the protein sequence evolution analysis, 12 genes (*rpl14*, *rpl16*, *rps4*, *rps15*, *rps18*, *rps19*, *psbK*, *psbN*, *ndhC*, *ndhJ*, *rpoB*, and *infA*) were detected under positive selection in branch of Sino-Vietnamese *Echinacanthus* species. These genes will lead to understanding the adaptation of *Echinacanthus* species to karst environment. The study will help to resolve the phylogenetic relationship and understand the adaptive evolution of *Echinacanthus*. It will also provide genomic resources and potential markers suitable for future species identification and speciation studies of the genus.

## Introduction

*Echinacanthus* Nees is a small genus in the tribe Ruellieae of the family Acanthaceae typified by *E. attenuatus* Nees (Nees Von Esenbeck, 1832; Anderson, 1867; Bentham, 1876; Bremekamp, 1965; Scotland and Vollesen, 2000; Deng et al., 2010; Hu et al., 2011; Tripp et al., 2013). The genus is characterized by anthers with spurred thecae and axillary or terminal inflorescence of thyrse type. Since its establishment, 16 species have been described within the genus, some of which had been later assigned to other genera (Nees Von Esenbeck, 1847; Anderson, 1867; Clarke, 1885; Kuntze, 1891; Lindau, 1895; Backer and Bakhuizen van den Brink, 1965; Wood, 1994). Lo and Fang (1985) described three new species from China, *E. flaviflorus* H. S. Lo and D. Fang, *E. longipes* H. S. Lo and D. Fang, and *E. longzhouensis* H. S. Lo. Later, Wood (1994) merged *E. flaviflorus* with *E. lofouensis* (H. Léveillé) J. R. I. Wood. At present, four species, *E. attenuatus*, *E. longipes*, *E. longzhouensis* and *E. lofouensis*, are recognized in the genus (Deng et al., 2010; Hu et al., 2011; Tripp et al., 2013). Initially, *Echinacanthus* was placed in the tribe Ruellieae (Nees Von Esenbeck, 1832) and adopted by Anderson (1867) and other researchers (Bentham, 1876; Bremekamp, 1965; Scotland and Vollesen, 2000; Hu et al., 2011; Tripp et al., 2013). However, the status of *Echinacanthus* within Ruellieae has been controversial. Bentham (1876) placed it in the subtribe Ruellieae Lindau (1895)considered it a member of the subtribe Strobilanthinae, whereas Bremekamp (1965) transferred *Echinacanthus* to the subtribe Petalidiinae. A recent phylogenetic study indicated that *E. attenuatus* belonged to the subtribe Petalidiinae, and the other three *Echinacanthus* species continued to be questioned (Tripp et al., 2013). Hence, the phylogeny of *Echinacanthus* in Ruellieae is not resolved. Furthermore, the phylogenetic relationships among the four species of *Echinacanthus* based on molecular markers have been incomplete because they did not include all the species of *Echinacanthus* (Gao, 2010; Tripp et al., 2013). Therefore, previous studies did not appear to fully resolve the phylogenetic relationship of *Echinacanthus*.

In addition, the four recognized species of *Echinacanthus* have special geographical distribution. *E. attenuatus* is restricted to the West Himalayas in Bhutan, India, and Nepal, while the remaining species are endemic to the Sino-Vietnamese karst region in southern China and northern Vietnam (Wood, 1994; Deng et al., 2010; Hu et al., 2011). Furthermore, the three Sino-Vietnamese species are typical for karst, and they are narrow endemics with almost non-overlapping areas. For example, *E. longipes* is widely distributed in southeastern Yunnan, southwestern Guangxi, and northern Vietnam, whereas, *E. lofouensis* is scattered in the border area between Guangxi and Guizhou provinces. In contrast, *E. longzhouensis* has a very narrow distribution as it only found in Longzhou in Guangxi province and Yangchun in Guangdong province. Despite it provides an important model for understanding the role of the Himalayas and limestone karst in speciation events and endemism, evolutionary history and speciation of *Echinacanthus* remain unclear. Hence, developing molecular markers and examining divergence regions will enable studies on speciation of *Echinacanthus* and adaptive evolution in the Sino-Vietnamese karst region.

Chloroplast is an important organelle that can transform the light energy into chemical energy in green plants (Ris and Plaut, 1961; Daniell et al., 2016). It has independent genome (chloroplast DNA, cpDNA) that is characterized by small molecular weight, multiple copies, and simple structure (Olmstead and Palmer, 1994; Wicke et al., 2011). With the development of high-throughput sequencing technologies, cpDNA has been widely utilized for reconstructing phylogenetic relationships, DNA barcoding, and development of molecular markers (Graham and Olmstead, 2000; Dong et al., 2014; Lemieux et al., 2016; Raman et al., 2016;Li Y.G. et al., 2017; Ng et al., 2017; Song et al., 2017; Zhang et al., 2017). Until now, the complete chloroplast genomes have been reported for more than 1,000 species, excluding *Echinacanthus*^1^. Although Acanthaceae is a large family consisting of 220–250 genera and 2,500–4,300 species, the complete chloroplast genomes have been sequenced for only three species (*Andrographis paniculata* (Burm.f.) Nees, NC_022451; *Ruellia breedlovei* T. F. Daniel, KP300014; *Strobilanthes cusia* (Nees) O. Kuntze, MG874806).

In the present study, we sequenced, characterized, and compared the complete chloroplast genomes of the four *Echinacanthus* species. This is the first comprehensive cp genomes of *Echinacanthus* species. Our main objectives were to: (1) deeply understanding the interspecific variation within the *Echinacanthus* cp genomes, (2) resolve phylogenetic relationships among the four species in *Echinacanthus* using the cp genome sequences, (3) examine simple sequence repeats (SSRs) and hotspot regions as candidate sequences for species identification and future speciation studies in *Echinacanthus*, and (4) identify genes underlying positive selection as potentially genes for adaptive evolution in karst region.

## Materials and Methods

### Plant Materials and DNA Extraction

Plant samples were collected in their native habitats and the voucher specimens were deposited in the South China Botanical Garden, Chinese Academy of Sciences (IBSC), and Royal Botanic Garden Edinburgh (E) (Table 1). Total genomic DNA was extracted from 100 mg silica gel-dried leaves following the method of CTAB (Doyle and Doyle, 1990).

**Table 1:** The basic characteristics of the chloroplast genomes of four *Echinacanthus* species.

Characteristics	*Echinacanthus longipes*	*Echinacanthus lofouensis*	*Echinacanthus longzhouensis*	*Echinacanthus attenuatus*
Location	Hekou, Yunnan, China	Libo, Guizhou, China	Yangchun, Guangdong, China	Phidim, Mechi, Nepal
Longitude	22.6858	25.3234	22.1874	27.14
Latitude	104.0199	108.0814	111.7408	87.7658
Voucher	F. Peng P16102201(IBSC)	Y. F. Deng 26020 (IBSC)	Y. Tong and F. Peng 14082413 (IBSC)	Adhikari B. and Kandel D. R.79 (E)
LSC length (bp)	83,875	82,561	83,947	83,610
SSC length (bp)	17,389	17,398	17,572	17,740
IR length (bp)	25,690	25,687	25,433	25,661
Total length (bp)	152,644	151,333	152,384	152,672
Protein-coding genes	80	80	80	80
tRNA genes	29	29	29	29
rRNA genes	4	4	4	4
Total number of genes	113	113	113	113
GC content (%)	38.62%	38.74%	38.64%	38.26%
Clean reads	2030586	1806880	1422624	1526374
Clean base	46145070000	41061352500	3232912500	34686855000
Read length (bp)	150 bp	150 bp	150 bp	150 bp


*LSC, large single copy; SSC, small single copy; IR, inverted repeat.*

### Genome Sequencing, Assembling, and Annotation

The chloroplast genome was amplified in overlapping fragments according to the methods described by Yang et al. (2014) at Germplasm Bank of Wild Species, Kunming Institute of Botany, Chinese Academy of Sciences. DNA samples were sheared into fragments of about 500 bp and used to construct libraries following the manufacturer’s instructions (Illumina, San Diego, CA, United States). Paired-end sequencing was conducted on Illumina HiSeq X-Ten platform. Raw reads were quality trimmed and the clean data were assembled after removing adapters using CLC Genomic Workbench v10 (CLC Bio., Aarhus, Denmark). Moreover, the raw sequencing data had been deposited in SRA (PRJNA504924). And then, the contigs were checked using BLAST searches^2^ against the available complete chloroplast sequence of *A. paniculata* (NC_022451). Relative position and direction of each contigs were manually adjusted according to the reference genome. Finally, the complete chloroplast genome was acquired in Geneious v.8.1 (Kearse et al., 2012). Annotation of the chloroplast genome was conducted using OGDRAW (Lohse et al., 2013). The genome map of the species was illustrated with the help of CPGAVAS (Liu et al., 2012), and the annotated chloroplast genome sequences were submitted to NCBI under accession numbers: MF490441, MH045155, MH045156, and MH045157.

### Repeat Sequence Analysis

We identified simple sequence repeats of *Echinacanthus* with MISA (Thiel et al., 2003) by setting the minimum number of repeats to 10, 5, 4, 3, 3, and 3 for mono-, di-, tri-, tetra-, penta-, and hexanucleotides, respectively.

### Genome Comparison and Analysis

The complete chloroplast genomes of the four species were compared using the program mVISTA (Frazer et al., 2004). DnaSP v.5.0 (Librado and Rozas, 2009) was employed to analyze nucleotide variability among the four species of *Echinacanthus*. We also compared the borders of large single copy (LSC), small singles copy (SSC), and inverted repeat (IR) regions in the genomes of the four species.

### Phylogenomic Analysis

Three chloroplast genomes of Acanthaceae (*A. paniculata*, NC_022451; *R. breedlovei*, KP300014; *S. cusia*, MG874806) and four outgroups in Lamiales (*Erythranthe lutea* (L.) G. L. Nesom, NC_030212; *Scrophularia dentata* Royle ex Benth., KT428154; *Tanaecium tetragonolobum* (Jacq.) L. G. Lohmann, KR534325; *Sesamum indicum* L., NC_016433) were downloaded from GenBank. The chloroplast genomes of these species and *Echinacanthus* species were aligned with MAFFT v.7 (auto strategy) (Katoh and Standley, 2013). The data matrix was subjected to Bayesian analysis using MrBayes v.3.2 (Ronquist et al., 2012). Before Bayesian analysis, TVM+I+G model was selected using program modeltest 3.7 under the Akaike Information Criterion (AIC) (Posada and Crandall, 1998). All parameters were set according to the chosen model as follow: statefreqpr = fixed (0.3112, 0.1886, 0.1829, 0.3173) revmat = fixed (0.9096, 1.7823, 0.4160, 1.0306, 1.7823, 1.000), shapepr = fixed (0.8693), apinvar = fixed (0.3286). The analysis, implementing Markov chain Monte Carlo (MCMC) algorithm, started from random trees. It was sampled every 100 generation and ran for 1,000,000 generations with four incremental heated chains. The first 1,000 trees corresponding to the “burn-in” period were discarded, and the remaining trees were used to construct the majority-rule consensus tree. Posterior probabilities (PP) > 0.95 were considered significant support for a clade. Maximum parsimony analysis was run in Paup^∗^ v.4.0b10 (Swofford, 2003). All characters were equally weighted and unordered. Heuristic search was performed with 1,000 replicates of random addition sequence, tree bisection-reconnection (TBR) branch swapping, retaining up to 10 most parsimonious trees at each replicate, and random addition of sequences with 100 replicates. Branches of zero length were collapsed and all multiple parsimonious trees were saved. Strict consensus trees were constructed from the most parsimonious trees. Bootstrap analyses were used to evaluate the support for individual clades (Felsenstein, 1985) with 1,000 replicates. Branches receiving bootstrap values (BS) > 70% were considered well supported (Hillis and Bull, 1993).

### Positive Selection Analysis of Protein Sequence

In order to detect the protein-coding genes under selection in Acanthaceae, the sequences for each gene were aligned separately and the maximum likelihood phylogenetic tree based on complete chloroplast was reconstructed using Paup^∗^ v.4.0b10 (Swofford, 2003). The synonymous (dS), non-synonymous (dN) nucleotide substitution rates and the dN/dS ratio (ω) were calculated using the codeml in Paml4.7 (Yang, 2007) with branch test model (Nielsen and Yang, 1998). Before analyses, the clade of Sino-Vietnamese species were set as foreground clade, and the others were set as background clade. The options of the two analyses were set to seqtype = 1, NSites = 0, model = 0 or model = 2. The likelihood ratio test (LRT) was used to estimate the quality of each model (Yang and Nielsen, 2002).

## Results

### Features of the Chloroplast Genome

**FIGURE 1:**
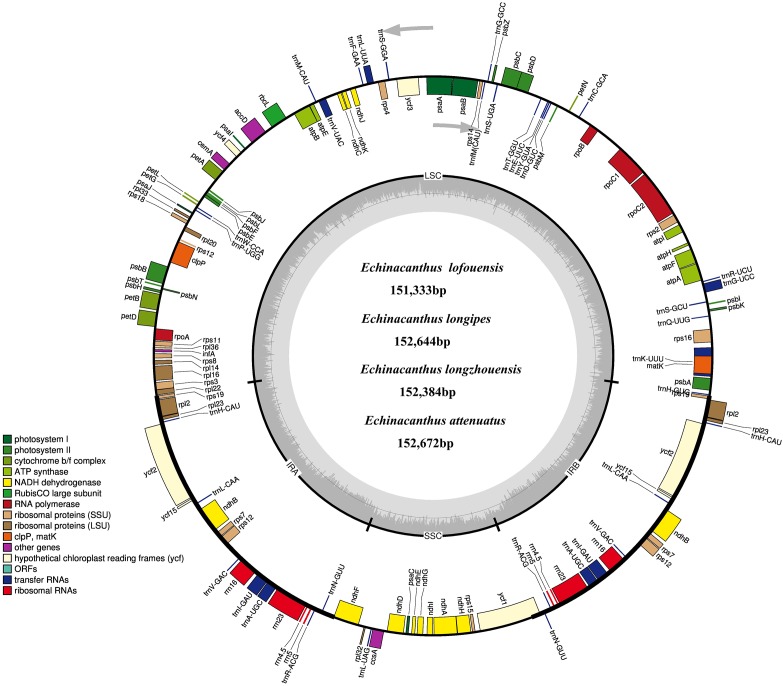
The chloroplast genome map of four *Echinacanthus* species. Genes lying outside the circle are transcribed in the counter clockwise direction, while those inside are transcribed in clockwise direction. The colored bars indicted different functional group. The darker gray area in the inner circle denoted GC content while the lighter gray corresponds to the AT content of the genome. LSC, large single copy; SSC, small single copy; IR, inverted repeat.

After removing low-quality reads and adapter sequences, 1,422,624–1,806,880 clean reads (150 bp average read length) were obtained for the four species. Through *de novo* assembly, contigs mapped to the closest species reference (*A. paniculata*) were then used for reconstructing the *Echinacanthus* cpDNA. The complete chloroplast genomes of the four species of *Echinacanthus* ranged from 151,333 bp (*E. lofouensis*) to 152,672 bp (*E. attenuatus*) with 38.26–38.74% GC content (Table 1). They had a typical circular structure with four junction regions: a separate LSC region of 82,561–83,947 bp, a SSC region of 17,398–17,740 bp, and a pair of IRs (IRa, IRb) each 25,433–25,690 bp (Table 1 and Figure 1). A total of 113 unique genes, comprising 80 protein-coding genes, 29 tRNA genes, and 4 rRNA genes, were detected in each *Echinacanthus* cpDNA (Table 2). The gene number, order, and name were very similar among the four *Echinacanthus* species (Figure 1). The seven protein-coding genes, seven tRNA genes, and four rRNA genes distributed in the two IR regions were present in two copies (Table 2). Of the 113 distinct genes, 18 genes contained one intron (15 genes) or two introns (*ycf3*, *clpP*, *rps12*) in the four chloroplast genomes. The *rps12* gene in *Echinacanthus* was recognized as trans-spliced gene, with the first exon located in the LSC region and the other two exons distributed in the IR regions. We also detected eight genes with partially overlapping sequences: *trnK*-UUU/*matK*, *atpE*/*atpB*, *psbD*/*psbZ*, and *rps3*/*rpl22*. Owing to the presence of internal stop codons, the genes *ycf1* and *ycf15* were identified as pseudogenes in *E. longipes* and *E. attenuatus*, respectively, while, *ycf2* and *rps19* were recognized as pseudogenes in *E. lofouensis*. In *E. longzhouensis*, *rps19* located in the IRb/LSC junction region was identified as pseudogene because of incomplete gene duplication.

**Table 2:** Gene contents in four *Echinacanthus* species chloroplast genome.

Classification	Genes
**Genetic apparatus**	
Large ribosomal subunits	*rpl2*^∗^(×2)*, rpl14*, *rpl16*^∗^, *rpl20*, *rpl22*, *rpl23*(×2), *rpl32*, *rpl33*, *rpl36*
Small ribosomal subunits	*rps2*, *rps3*, *rps4*, *rps7* (×2), *rps8*, *rps11*, *rps12*^∗∗a^, *rps14*, *rps15*, *rps16*^∗^, *rps18*, *rps19*(×2)
DNA dependent RNA polymerase	*rpoA*, *rpoB*, *rpoC1*^∗^, *rpoC2*
Protease	*clpP*^∗∗^
Maturase	*matK*
Ribosomal RNAs	*rrn4.5*(×2)*, rrn5*(×2)*, rrn23*(×2)*, rrn16*(×2)
Transfer RNAs	*trnA-*UGC(×2)^∗^, *trnC-*GCA, *trnD-*GUC, *trnE-*UUC, *trnF-*GAA, *trnfM-*CAU, *trnG-*GCC, *trnG-*UCC^∗^, *trnH-*CAU(×2), *trnH-*GUG, *trnI-*GAU(×2)^∗^, *trnK-*UUU^∗^, *trnL-*CAA(×2), *trnL-*UAG, *trnL-*UUA^∗^, *trnM-*CAU, *trnN-*GUU(×2), *trnP-*UGG, *trnQ-*UUG, *trnR-*ACG(×2), *trnR-*UCU, *trnS-*GCU, *trnS-*GGA, *trnS-*UGA, *trnT-*GGU, *trnV-*GAC(×2), *trnV-*UAC^∗^, *trnW-*CCA, *trnY-*GUA
**Light dependent photosynthesis**	
Photosystem I	*psaA*, *psaB*, *psaC*, *psaI*, *psaJ*
Photosystem II	*psbA*, *psbB*, *psbC*, *psbD*, *psbE*, *psbF*, *psbH*, *psbI*, *psbJ*, *psbK*, *psbL*, *psbM*, *psbN*, *psbT*, *psbZ*
NAD(P)H dehydrogenase complex	*ndhA*^∗^, *ndhB*^∗^(×2), *ndhC*, *ndhD*, *ndhE*, *ndhF*, *ndhG*, *ndhH*, *ndhI*, *ndhJ*, *ndhK*
F-type ATP synthase	*atpA*, *atpB*, *atpE*, *atpF*^∗^, *atpH*, *atpI*
Cytochrome b6/f complex	*petA*, *petB*^∗^, *petD*^∗^, *petG*, *petL*, *petN*
**Light independent photosynthesis**	
Inner membrane protein	*cemA*
Cytochrome c biogenesis protein	*ccsA*
Large subunit of Rubisco	*rbcL*
Subunit of acetyl-CoA-carboxylase	*accD*
Translation initiation factor	*infA*
**Function uncertain**	*ycf1*, *ycf2*(×2), *ycf3*^∗∗^, *ycf4*, *ycf15*(×2)


*^∗^Gene containing one intron, ^∗∗^ gene containing two introns, ^a^ trans-splinting gene, (×2) shows genes have two copies.*

### Simple Sequence Repeats Analysis

**FIGURE 2:**
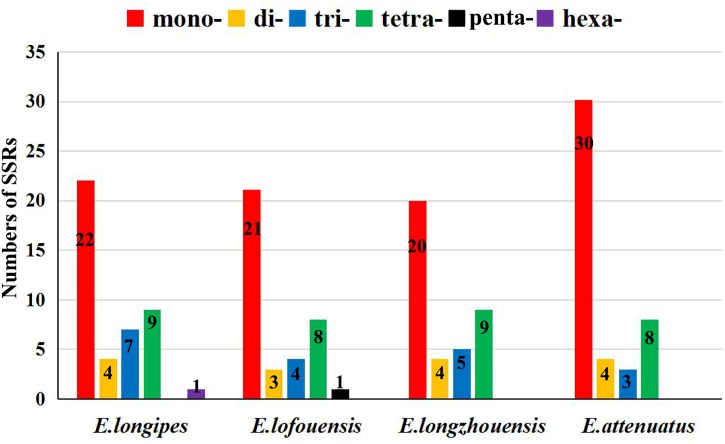
The numbers of the simple sequence repeats (SSRs) in the chloroplast genomes of four *Echinacanthus* species. mono-, mononucleotides; di-, dinucleotides; tri-, trinucleotides; tetra-, tetranucleotides; penta-, pentanucleotides; hexa-, hexanucleotides.

A total of 37–45 SSR loci were discovered in the four chloroplast genomes. Mono-, di-, tri-, and tertranucleotide SSRs were all detected in the four species (Figure 2). Additionally, pentanucleotide and hexanucleotide SSRs were found in *E. lofouensis* and *E. longipes*, respectively. In the four species, more than half of the SSRs were mononucleotide repetitions (51.16%, 56.76%, 52.63%, and 66.67% in *E. longipes*, *E. lofouensis*, *E. longzhouensis*, and *E. attenuatus*, respectively). Meanwhile, of the detected SSR regions, 86 SSRs were identified in intergenic spaces, while 51 SSRs were located in the coding DNA sequence and 26 SSRs in the coding sequence introns (Supplementary Table S1). Interestingly, all dinucleotide SSRs belonged to AT type and the majority of mononucleotide, trinucleotide, tetranucleotide, pentanucleotide, and hexanucleotide SSRs were especially rich in A or T (Supplementary Table S1).

### Comparative Genomic Analysis

**FIGURE 3:**
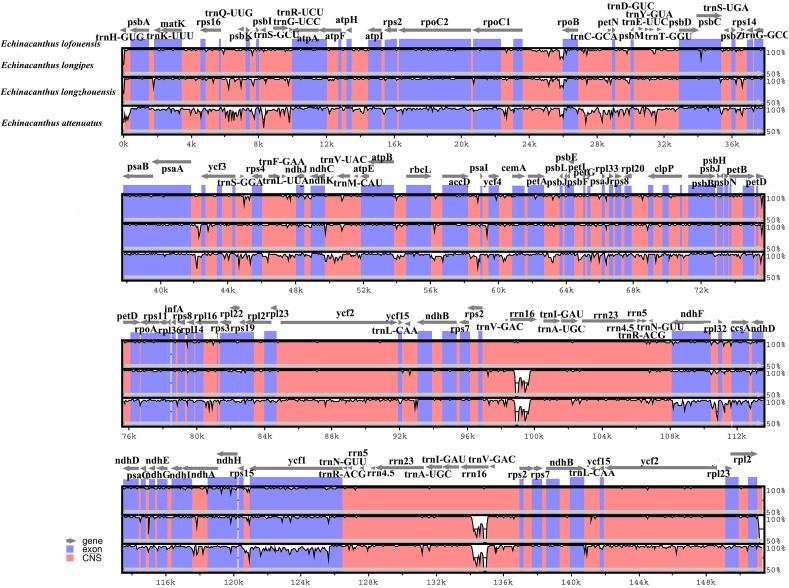
Comparison of four *Echinacanthus* chloroplast genomes using mVISTA program, taking the annotation of *E. lofouensis* as a reference. The top line shows the genes in order. A cut-off of 70% identity was used for the plots and the Y-scale represents the percent identity between 50 and 100%. Genome regions are color-coded as exon and conserved non-coding sequences (CNS).

We used program mVISTA to analyze the overall sequences of the four species using the annotated *E. lofouensis* sequence as a reference (Figure 3). A genome alignment revealed an overall high sequence similarity among the four species. In addition, LSC and SSC regions were more divergent compared with IR regions. The regions with highest level of divergence were *rrn16*, *ndhF*, *ndhA*, *ycf1*, *psbA*-*trnH*, *accD*-*psaI*, *rps16*-*trnQ*-UUG, *trnS*-GCU-*trnG*-UCC, *rpoB*-*trnC*-GCA, *psaA*-*ycf3*, *trnS*-GGA-*rps4*, *ndhF*-*rpl32*, and *rps15*-*ycf1*. Furthermore, the comparative genomic analyses showed lower sequence divergence between Sino-Vietnamese *Echinacanthus* species. The highest level of divergence across them was detected in *rrn16*, *ndhA*, *ycf1*, *rps16*-*trnQ-*UUG, and *psaA*-*ycf3*.

**FIGURE 4:**
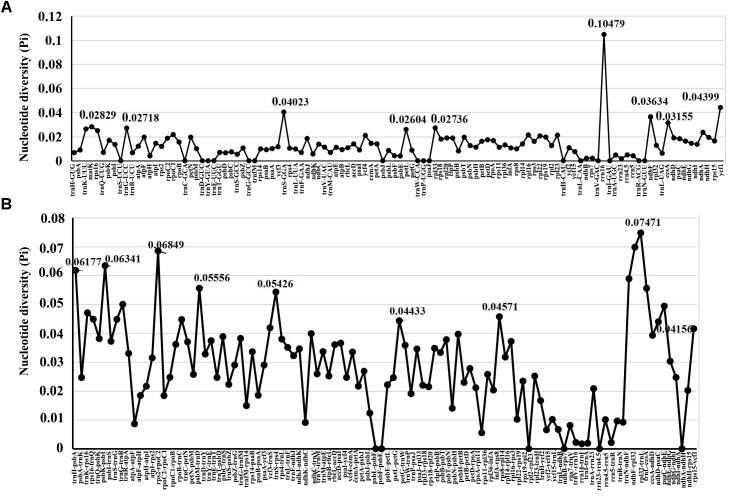
Comparative analysis of the nucleotide diversity (Pi) values among four *Echinacanthus* chloroplast genomes. **(A)** Coding regions. **(B)** Non-coding regions. *X*-axis: name of the regions; *Y*-axis: nucleotide diversity (Pi).

For the further understanding the sequence divergence among these four *Echinacanthus* species, the coding regions and intergenic regions were extracted to calculate nucleotide variability (Pi) (Supplementary Table S2 and Figure 4). The Pi value ranged from 0 to 0.10479. The coding regions were also more conserved compared to the intergenic spacer. Meanwhile, the LSC and SSC regions were much more divergent than the IR region. However, the highest nucleotide diversity (Pi = 0.10479, *rrn16*) was present in the IR region. A total of 22 regions, *trnS*-*GGA*, *rrn16*, *ycf1*, *trnH*-*psbA*, *trnK*-UUU-*rps16*, *rps16-trnQ*-UUG, *psbK-psbI*, *trnS*-GCU-*trnG*-UCC, *rps2-rpoC2*, *trnC*-GCA-*petN*, *trnD*-GUC-*trnY*-GUA, *ycf3-trnS*-GGA, *trnS*-GGA-*rps4*, *petG-trnW*-CCA, *rps8-rpl14*, *ndhF-rpl32*, *rpl32-trnL*, *trnL*-UAG-*ccsA*, *ccsA-ndhD*, *ndhC-ndhE*, *ndhE-ndhG*, and *rps15-ycf1* were recognized as hotspot regions with nucleotide diversity > 0.04. Of those, 13 regions were located in the LSC, one in the IR, and eight in the SSC region (Supplementary Table S2).

**FIGURE 5:**
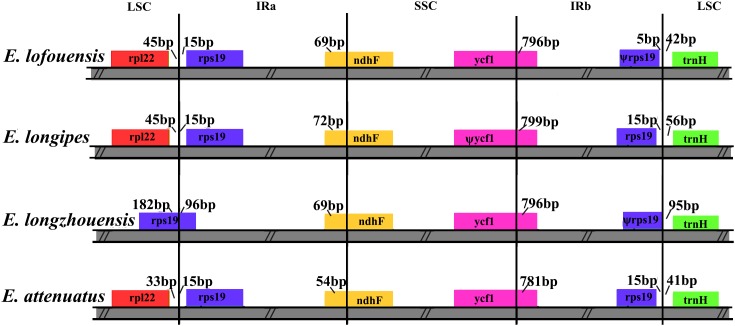
Comparison of the LSC, IR, and SSC junction regions among four *Echinacanthus* chloroplast genomes. Genes are denoted by colored boxes. Ψ shows the pseudogene. The numbers above the gene features indicates the distance between the end of the gene and the borders sites. The slashes indicate the location of the distance. This figure is not to scale.

The expansion and contraction of the border regions were analyzed for the four *Echinacanthus* species (Figure 5). The genes *rpl22*, *rps19*, *ndhF*, *ycf1*, and *trnH* were present at the junction of the LSC/IRa, IRa/SSC, SSC/IRb, and IRb/LSC borders. The LSC/IRa junction regions were relatively stable in *E. lofouensis*, *E. longipes*, and *E. attenuatus.* There were 33–45 bp between *rpl22* and the LSC/IRa border, meanwhile, the *rps19* generated a distance of 15 bp to another LSC/IRa junction. In contrast, there were 96 bp protrusion of *rps19* gene into IRa region in *E. longzhouensis*. Moreover, the IRa/SSC borders were well conserved among the four cp genomes, of which the IRb region expanded into the gene *ndhF* with 54–72 bp. The *ycf1* gene crossed the SSC/IRb junction extending 796–799 bp to IRb regions. In addition, the distance between *trnH*-GUG and IRb/LSC border for all the four species varied from 41 to 95 bp. On the other hand, although the *rps19* gene was located in the IRb/LSC border, the exception was *E. longzhouensis*, in which the ψ*rps19* gene was incomplete duplicated.

### Phylogenetic Analysis

**FIGURE 6:**
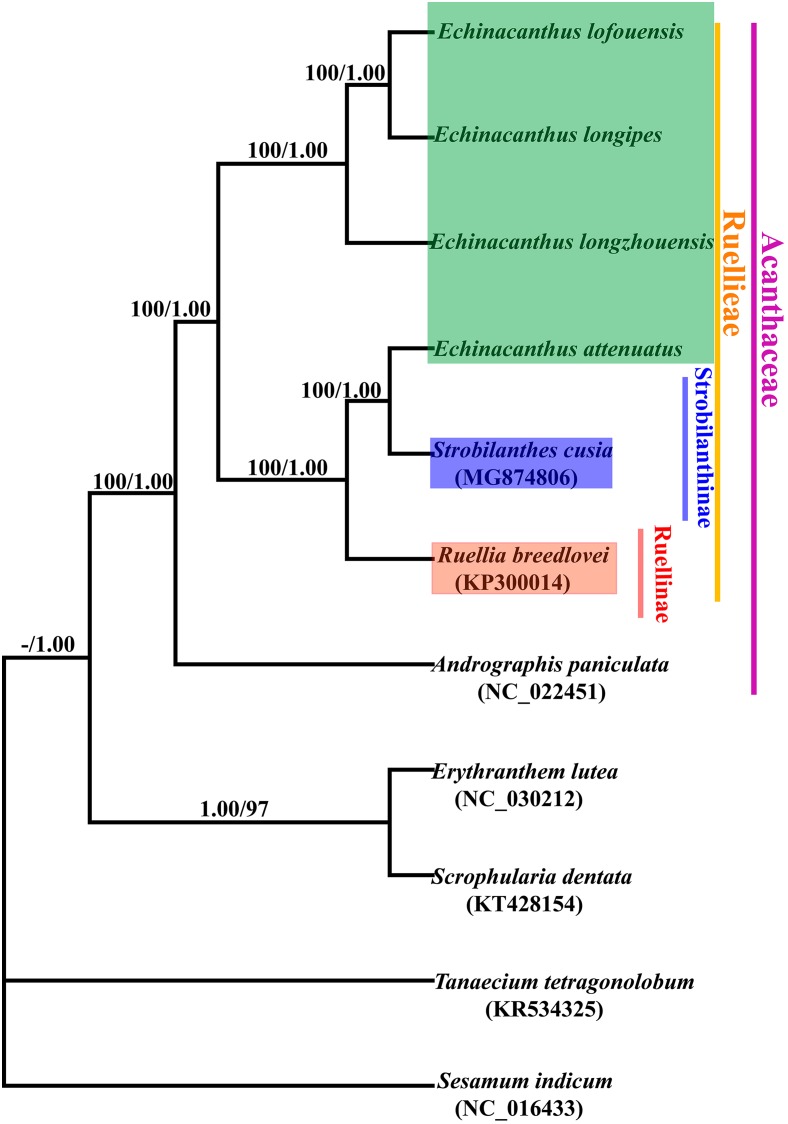
Phylogenetic tree conducted using Bayesian Inference (BI) and most parsimonious (MP) methods based on whole complete genomes from different species. The numbers above branches represent posterior probability (PP)/bootstrap percentage (BP).

In order to resolve the phylogenetic relationships of *Echinacanthus*, the complete chloroplast genomes of seven species in Acanthaceae and four outgroups were used to build the phylogenetic trees. In our analysis, the tree topologies of the datasets based on the parsimony and Bayesian analyses were highly congruent (Figure 6). All the sampled species of Acanthaceae were clustered into one clade with 100% bootstrap value or the Bayesian posterior probability. Moreover, three major clades were identified in Acanthaceae and the analyses obtained high support for all of the three nodes. At the same time, *Echinacanthus* species were segregated into two clades. *E. lofouensis*, *E. longipes*, and *E. longzhouensis* were in a well-supported clade (PP = 1.00, BS = 100), with *E. longzhouensis* being the earliest diverging lineage. *E. attenuatus* and *S. cusia* formed a clade, which was identified as a sister to *R. breedlovei* with high support value (PP = 1.00, BS = 100).

### Positive Selection Analysis of Protein Sequence

In order to assess the selective pressure on Sino-Vietnamese karst species of *Echinacanthus* in Acanthaceae, the branch model was used to examine the genes under positive selection. In the present study, the null hypothesis was that all the branches had the same ω and the alternative hypotheses was that the foreground clade had different ω. Then, based on the LRT, *P*-value > 0.05 denoted the alternative hypothesis and *P*-value < 0.05 represented the null hypothesis. Moreover, the ratio of dN/dS > 1 indicated that the genes were under positive selection. dN/dS < 1 was suggested that the genes were under negative selection. dN/dS = 1 indicated neutral selection. Thus, we found that 12 genes including *ndhC*, *ndhJ*, *psbK*, *psbN*, *rpl14*, *rpl16*, *rps4*, *rps15*, *rps18*, *rps19*, *infA*, and *rpoB* were under positive selection in the branch of Sino-Vietnamese karst species (Supplementary Table S3).

## Discussion

### Chloroplast Sequence Variation

The analysis of the chloroplast genomes of the four *Echinacanthus* species presented herein revealed a typical quadripartite structure, with a pair of IR regions, a single LSC region, and a single SSC region. The size of the genome ranged from 151,333 (*E. lofouensis*) to 152,672 bp (*E. attenuatus*), which was longer than the genomes of *A. paniculata* (Ding et al., 2016), *R. breedlovei*, and *S. cusia* (Chen et al., 2018). In addition, the four species shared similar GC content (about 38%). In particular, *rps12* in *Echinacanthus* was recognized as the trans-spliced gene, which was in line with observations in other species (Hildebrand et al., 1988; Liu et al., 2016; Gichira et al., 2017; Chen et al., 2018). The overall gene contents and arrangement were similar among the four *Echinacanthus* species. Nevertheless, comparative genome analysis with mVISTA revealed the DNA sequences divergence among related species. The comparison showed that the cp genomes among *E. longipes*, *E. longzhouensis*, and *E. lofouensis* were highly conserved. Interestingly, we observed that *E. attenuatus* had greater difference with the other three species. Our results had confirmed the differentiation of the geographic distribution.

Another remarkable variation among the four *Echinacanthus* species was the location of the boundaries between the four chloroplast regions. Location of the boundaries, especially of the contraction and expansion of the regions, sheds light on the evolution of taxa (Nazareno et al., 2015; Menezes et al., 2018). As a typical chloroplast genome structure, the IR/LSC boundaries in *E. longzhouensis* expand into *rps19*. This expansion may be an ancestral symplesiomorphy in Liliaceae (Li P. et al., 2017). In the present phylogenetic study, although *E. longzhouensis* was not the basal species of *Echinacanthus*, it was basal in the Sino-Vietnamese *Echinacanthus* clade. Thus, the result may provide new insight into the evolution of *Echinacanthus* species from the Sino-Vietnamese karst region. Furthermore, LSC/IR regions of the chloroplast genome in Lamiales were divided into four different types (Chen et al., 2018). Only type II, which included an *rps19* pseudogene, was found in the chloroplast genome of *Echinacanthus*. As the duplicated complement of *rps19* was included in the IR regions, the fifth type of the LSC/IR region was suggested in Lamiales.

### Phylogenetic Analysis

The genus *Echinacanthus* was placed in the tribe Ruellieae of Acanthaceae (Nees Von Esenbeck, 1847; Bentham, 1876; Bremekamp, 1965; Scotland and Vollesen, 2000; Hu et al., 2011; Tripp et al., 2013). Previous molecular research identified seven subtribes within Ruellieae: Erantheminae, Ruellinae, Trichantherinae, Hygrophilinae, Mimulopsinae, Petalidiinae, and Strobilanthinae (Tripp et al., 2013). *Echinacanthus* was initially placed in the subtribe Ruellinae, but its status had long been controversial (Bentham, 1876; Lindau, 1895; Hu et al., 2011) and another treatment placed it within taxa with uncertain position (Tripp et al., 2013). The present phylogenetic analyses based on seven Acanthaceae taxa confirmed the monophyly of the family as previously reported (Chen et al., 2018). Furthermore, all of the six Ruellieae taxa formed a monophyletic group. However, our phylogenetic analyses did not resolve *Echinacanthus* monophyletic within Ruellieae. Instead, *Echinacanthus* was divided into two groups. Thus, the two groups were named the West Himalayan group and the Sino-Vietnamese group. In this study, the West Himalayan group, which was previously placed in Petalidiinae based on morphology (Tripp et al., 2013), was closely related to *Strobilanthes* of Strobilanthinae. In contrast, the Sino-Vietnamese species formed a highly supported distantly related group. Our previous molecular studies allied the Sino-Vietnamese *Echinacanthus* plants with *Eranthemum*, *Pararuellia*, and *Leptosiphonium*. They were placed within Erantheminae with a high support value (Gao, 2010). Also, the two geographical groups are clearly differentiated by the type of inflorescence, i.e., the West Himalayan species has thyrsus, while the Sino-Vietnamese plants have a cyme. Hence, the Sino-Vietnamese *Echinacanthus* should be recognized as a new genus. But, the positions of the two group of *Echinacanthus* in Ruellieae still remain somewhat uncertain and the future taxonomic analyses should incorporate additional chloroplast genomes of the Acanthaceae. But beyond that, the relationships within *Echinacanthus* species were fairly well resolved in this study, especially the Sino-Vietnamese *Echinacanthus* species. In fact, the three Sino-Vietnamese species were similar to one another in morphology. *E. lofouensis* and *E. longzhouensis* were shrubs, but, *E. longipes* was the perennial herbs. Furthermore, *E. longzhouensis* and *E. longipes* shared the same traits with purple corolla and two aristate appendages at the base of each thecous. The current phylogenetic tree showed the deep-level relationships of Sino-Vietnamese *Echinacanthus* species. It was revealed that *E. longipes* had a closer phylogenetic relationship to *E. lofouensis* than to *E. longzhouensis.* The current phylogenetic analysis raise the possibility that cp genomes may be useful for studying phylogeny and speciation of *Echinacanthus* in the future.

### Molecular Markers and Hotspot Regions Identification

Simple sequence repeats are shortly repeated DNA sequence motifs consisting of repeat units of 1–6 bp in length. As genetic markers, they are widely dispersed in genomes. The advantages of SSRs include high polymorphism, high abundance, codominance, selective neutrality and the possibility of antomated detection and scoring. They had been extensively used in population genetics and molecular evolution studies (George et al., 2015; Govindaraj et al., 2015). In the present study, the results were the same as most other species, for example, the majority of SSRs were mononucleotide repeats, located in intergenic spacer regions, and contributed to AT richness (Morton et al., 1997; Xu et al., 2017). SSRs are effective molecular markers for interspecific polymorphisms. According to the criteria of SSRs, i.e., presence of the same repeat units, being located in the homologous regions, and having different number of the repeat units, 22 SSRs were identified as polymorphic SSRs between *Echinacanthus* species (Table 3). In addition, our alignment and nucleotide diversity revealed high level of similarity across the four species. Similar to most other species, the IR regions were more conserved than the LSC and SSC regions, whereas the coding regions were less divergent than the non-coding regions (Shivakumar et al., 2017; Zeng et al., 2017). The divergence level between the two geographic groups was consistent with the phylogenetic relationships of the genus. According to the analyses of sequence divergence conducted with mVISTA and nucleotide variability inferred by DnaSP, the top six most divergent regions between *Echinacanthus* species were *rrn16*, *ycf1*, *ndhA*, *rps16*-*trnQ*-UUG, *trnS*-GCU-*trnG*-UCC, and *psaA*-*ycf3*. Above all, SSRs and the hotspot regions can be used as candidate DNA barcodes in phylogenetic, plant identification and speciation studies in *Echinacanthus*.

**Table 3:** The polymorphic simple sequence repeats in *Echinacanthus.*

Type	*E. longipes*/*E. lofouensis*/ *E. longzhouensis*/*E. attenuatus*	Location	Regions
TA	5/5/4/2	*trnQ-*UUG*-psbK*	LSC
AT	5/5/5/4	*trnS-*GCU*-trnG-*UCC	LSC
TA	5/0/0/0	*psbC*	LSC
TA	3/0/3/5	*rps4-trnL-*UUA	LSC
AT	2/2/2/5	*rpoC2*	LSC
TTA	4/4/4/0	*rpoC1* intron	LSC
ATT	4/2/4/2	*trnV-*UAC intron	LSC
AAT	6/1/2/3	*petA-psbJ*	LSC
ATA	2/6/2/4	*petA-psbJ*	LSC
ATA	4/3/3/4	*trnP-*UGG*-psaJ*	LSC
TAT	4/4/4/2	*ycf1*	LSC
TCT	4/4/4/3	*ycf1*	LSC
AAGA	3/3/2/2	*clpP-psbB*	LSC
CAAT	3/3/3/2	*ndhA* intron	SSC
AATC	3/3/3/1	*rps15-ycf1*	SSC
ATAG	3/0/0/0	*rbcL-accD*	LSC
TTCT	2/2/3/2	*ndhI-ndhA*	SSC
AATT	2/2/2/3	*trnG-*GCC*-trnfM*	LSC
CAAA	2/2/2/3	*petA-psbJ*	LSC
TAAA	2/2/2/3	*trnP-*UGG*-psaJ*	LSC
TAGA	2/2/2/3	*trnH-*GUG*-psbA*	LSC
AATTAA	3/2/2/0	*rpoc1* intron	LSC


*LSC, large single copy; SSC, small single copy; IR, inverted repeat.*

### Adaptive Evolution Analysis

Acanthaceae is a tropical and subtropical family with high species, geographic, and ecological diversity (Borg et al., 2008; Daniel et al., 2008; Hu et al., 2011; Deng et al., 2010; Tripp and Siti, 2012; Tripp et al., 2017). To resolve the evolutionary history of its species, it is necessary to analyze the genetic diversity and adaptive evolution in Acanthaceae. The non-synonymous/synonymous rate ratio (ω = dN/dS) is very useful for measuring selective pressure at the protein level. The genes with positive selection played key roles in the adaptation to diverse environment (Fan et al., 2018; Wu et al., 2018). *Echinacanthus* contains two geographical groups with *E. longzhouensis*, *E. lofouensis* and *E. longipes* distributed in karst landform. Karsts are specific landforms which developed on soluble rocks such as limestone, marble and gypsum since Cambrian to the Quaternary ages (Ford and Williams, 2007). In this study, we examined the selective pressure of 80 protein genes in different branches of Acanthaceae to test adaptive genes of karst landform. As a result, 12 genes with dN/dS > 1 in branch of Sino-Vietnamese species were detected. Interestingly, of these 12 genes, six genes (*rpl14, rpl16, rps4, rps15, rps18, rps19*) have functions in chloroplast ribosome (Tiller and Bock, 2014). In addition, *psbK* and *psbN*, which have important role in photosystem, were detected under positive pressure in karst environment. Two NAD(P)H dehydrogenase complex genes were found under positive selection. Moreover, *rpoB* and *infA* were crucial for genetic information transmission, which affect transcription of DNA into RNA and translation of RNA to protein. They were also under adaptive selection in karst plant. These genes under positive selection may play an important role in the adaptation of *Echinacanthus* species to Sino-Vietnamese karst environment.

## Author Contributions

YD designed the experiments and contributed to the sampling. CG performed the experiments, analyzed the data, and wrote the manuscript. JW analyzed the data. All authors read and approved the final manuscript.

## Conflict of Interest Statement

The authors declare that the research was conducted in the absence of any commercial or financial relationships that could be construed as a potential conflict of interest.

## Footnotes

1 https://www.ncbi.nlm.nih.gov/genome

2 https://blast.ncbi.nlm.nih.gov/
