# Supplementary material for: The Complete Chloroplast Genomes of Echinacanthus Species (Acanthaceae): Phylogenetic Relationships, Adaptive Evolution, and Screening of Molecular Markers
Source: Front Plant Sci. 2019 Jan 10;9:1989. doi: 10.3389/fpls.2018.01989 (PMC6335349; doi:10.3389/fpls.2018.01989)
Supplement: Supplementary file 1 [file Table_1.DOCX]

Supplementary Material

**The Complete Chloroplast Genomes of *Echinacanthus* Species (Acanthaceae): Phylogenetic Relationships, Adaptive Evolution, and Screening of Molecular Markers**

**Chunming Gao^1, 2, 3^, Yunfei Deng^4, 5^, Jun Wang^1, 2, 3^**

*** Correspondence:**

Corresponding Author:

Yunfei Deng

No. 723, Xingke Lu, Tianhe Qu, Guangzhou, Guangdong, 510650, P.R.China

Email address: [yfdeng@scbg.ac.cn](mailto:yfdeng@scbg.ac.cn)

Table S1 Simple sequence repeats (SSRs) in the four *Echinacanthus* species*.*

| Species | Type | Repeat size (bp) | Start site (bp) | Location | Region |
| --- | --- | --- | --- | --- | --- |
| *Echinacanthus longipes* | A | 10 | 6707 | *rps16-trnQ-*UUG | LSC |
|  | T | 10 | 7814 | *psbK-psbI* | LSC |
|  | A | 12 | 7696 | *psbK-psbI* | LSC |
|  | A | 10 | 12232 | *atpF* intron | LSC |
|  | C | 11 | 16225 | *rps2-rpoC2* | LSC |
|  | T | 17 | 28508 | *trnC-*GCA*-petN* | LSC |
|  | A | 10 | 36335 | *psbZ-trnG-*GCC | LSC |
|  | C | 10 | 40397 | *psaA* | LSC |
|  | A | 11 | 46531 | *rps4-trnL-*UUA | LSC |
|  | T | 14 | 51667 | *ndhC-trnV-*UAC | LSC |
|  | T | 10 | 54693 | *atpB* | LSC |
|  | A | 10 | 68680 | *rps18-rpl20* | LSC |
|  | T | 10 | 78264 | *rpoA* | LSC |
|  | T | 10 | 84184 | *rps19-rpl2* | IR |
|  | T | 13 | 112049 | *ndhF-rpl32* | SSC |
|  | T | 10 | 112996 | *trnL-*UAG*-ccsA* | SSC |
|  | T | 11 | 124387 | *ycf1* | SSC |
|  | T | 10 | 124441 | *ycf1* | SSC |
|  | T | 11 | 124663 | *ycf1* | SSC |
|  | T | 10 | 124984 | *ycf1* | SSC |
|  | A | 10 | 126607 | *ycf1* | SSC |
|  | A | 10 | 152279 | *rpl2-rps19* | IR |
|  | TA | 10 | 6998 | *trnQ-*UUG*-psbK* | LSC |
|  | TA | 10 | 8333 | *trnS-*GCU*-trnG-*UCC | LSC |
|  | AT | 10 | 8361 | *trnS-*GCU*-trnG-*UCC | LSC |
|  | TA | 10 | 34260 | *psbC* | LSC |
|  | TTA | 12 | 22596 | *rpoC1* intron | LSC |
|  | TTC | 12 | 35333 | *psbC* | LSC |
|  | ATT | 12 | 51755 | *trnV-*UAC intron | LSC |
|  | AAT | 18 | 64178 | *petA-psbJ* | LSC |
|  | ATA | 12 | 67278 | *trnP-*UGG*-psaJ* | LSC |
|  | TAT | 12 | 124324 | *ycf1* | SSC |
|  | TCT | 12 | 124751 | *ycf1* | SSC |
|  | ATAA | 12 | 15429 | *atpI-rps2* | LSC |
|  | ATAG | 12 | 57565 | *rbcL-accD* | LSC |
|  | AAAC | 12 | 68236 | *rpl33-rps8* | LSC |
|  | TTTC | 12 | 71563 | *ClpP* intron | LSC |
|  | AAGA | 12 | 72276 | *clpP-psbB* | LSC |
|  | AATA | 12 | 114436 | *ndhD* | SSC |
|  | CAAT | 12 | 119552 | *ndhA* intron | SSC |
|  | AATC | 12 | 122021 | *rps15-ycf1* | SC |
|  | AATT | 12 | 123928 | *ycf1* | SSC |
|  | AATTAA | 18 | 15429 | *atpI-rps2* | LSC |
| *Echinacanthus lofouensis* | T | 10 | 7808 | *psbK-psbI* | LSC |
|  | A | 10 | 12187 | *atpF* intron | LSC |
|  | C | 10 | 16173 | *rps2-rpoC2* | LSC |
|  | A | 10 | 16188 | *rps2-rpoC2* | LSC |
|  | A | 10 | 28202 | *trnC-*GCA*-petN* | LSC |
|  | T | 17 | 28496 | *trnC-*GCA*-petN* | LSC |
|  | C | 10 | 40285 | *psaA* | LSC |
|  | T | 10 | 53749 | *atpB* | LSC |
|  | A | 10 | 53824 | *atpB-rbcL* | LSC |
|  | T | 10 | 68388 | *rpl20-rps12* | LSC |
|  | T | 11 | 69478 | *clpP* intron | LSC |
|  | A | 10 | 69627 | *clpP* intron | LSC |
|  | A | 10 | 70112 | *clpP* intron | LSC |
|  | T | 10 | 76929 | *rpoA* | LSC |
|  | T | 10 | 82842 | *rps19-rpl2* | IR |
|  | T | 15 | 110734 | *ndhF-rpl32* | SSC |
|  | T | 10 | 123140 | *ycf1* | SSC |
|  | T | 11 | 123361 | *ycf1* | SSC |
|  | T | 10 | 123682 | *ycf1* | SSC |
|  | A | 10 | 125305 | *ycf1* | SSC |
|  | A | 10 | 150976 | *rpl2-rps19* | IR |
|  | TA | 10 | 6984 | *trnQ-*UUG*-psbK* | LSC |
|  | TA | 10 | 8289 | *trnS-*GCU*-trnG-*UCC | LSC |
|  | AT | 10 | 8318 | *trnS-*GCU*-trnG-*UCC | LSC |
|  | TTA | 12 | 22535 | *rpoC1* intron | LSC |
|  | TTC | 12 | 35221 | *psbC* | LSC |
|  | TAT | 12 | 123022 | *ycf1* | SSC |
|  | TCT | 12 | 123449 | *ycf1* | SSC |
|  | ATAA | 12 | 15377 | *atpI-rps2* | LSC |
|  | AAAC | 12 | 66936 | *rpl33-rps8* | LSC |
|  | TTTC | 12 | 70251 | *clpP* intron | LSC |
|  | AAGA | 12 | 70983 | *clpP-psbB* | LSC |
|  | AATA | 12 | 113112 | *ndhD* | SSC |
|  | CAAT | 12 | 118223 | *ndhA* intron | SSC |
|  | AATC | 12 | 120719 | *rps15-ycf1* | SSC |
|  | AATT | 12 | 122626 | *ycf1* | SSC |
|  | TCAAT | 15 | 4223 | *trnK-*UUU*-rps16* | LSC |
| *Echinacanthus longzhouensis* | C | 11 | 16112 | *atpF* | LSC |
|  | T | 20 | 28379 | *trnC-*GCA*-petN* | LSC |
|  | T | 12 | 35409 | *psbC-trnS-*UGA | LSC |
|  | C | 11 | 36180 | *psbZ-trnG-*GCC | LSC |
|  | C | 10 | 40301 | *psaA* | LSC |
|  | T | 10 | 42388 | *psaA-ycf3* | LSC |
|  | A | 10 | 46364 | *rps4-trnL-*UUA | LSC |
|  | T | 10 | 54541 | *atpB* | LSC |
|  | A | 10 | 71239 | *clpP* intron | LSC |
|  | T | 10 | 78057 | *rpoA* | LSC |
|  | G | 10 | 103365 | *trnA-*UGC intron | IR |
|  | T | 10 | 111805 | *ndhF-rpl32* | SSC |
|  | T | 15 | 112256 | *rpl32-trnL-*UAG | SSC |
|  | A | 10 | 112282 | *rpl32-trnL-*UAG | SSC |
|  | T | 10 | 117433 | *ndhI* | SSC |
|  | T | 12 | 124543 | *ycf1* | SSC |
|  | T | 11 | 124617 | *ycf1* | SSC |
|  | T | 10 | 124936 | *ycf1* | SSC |
|  | A | 10 | 126559 | *ycf1* | SSC |
|  | C | 10 | 132770 | *trnA-*UGC intron | IR |
|  | AT | 10 | 6932 | *trnQ-*UUG*-psbK* | LSC |
|  | TA | 10 | 8233 | *trnS-*GCU*-trnG-*UCC | LSC |
|  | AT | 10 | 8261 | *trnS-*GCU*-trnG-*UCC | LSC |
|  | TA | 10 | 46230 | *rps4-trnL-*UUA | LSC |
|  | TTA | 12 | 22465 | *rpoC1* intron | LSC |
|  | TTC | 12 | 35174 | *psbC* | LSC |
|  | ATT | 12 | 51605 | *trnV-*UAC intron | LSC |
|  | TAT | 12 | 124204 | *ycf1* | SSC |
|  | TCT | 12 | 124703 | *ycf1* | SSC |
|  | ATAA | 12 | 15316 | *atpI-rps2* | LSC |
|  | AAAC | 12 | 68073 | *rpl33-rps8* | LSC |
|  | TTTC | 12 | 71378 | *clpP* intron | LSC |
|  | AGAA | 12 | 72112 | *clpP-psbB* | LSC |
|  | AATA | 12 | 114185 | *ndhD* | SSC |
|  | TTCT | 12 | 117968 | *ndhI-ndhA* | SSC |
|  | CAAT | 12 | 119404 | *ndhA* intron | SSC |
|  | AATC | 12 | 121891 | *rps15-ycf1* | SSC |
|  | AATT | 12 | 123799 | *ycf1* | SSC |
| *Echinacanthus attenuatus* | T | 11 | 3096 | *trnK-*UUU | LSC |
|  | A | 13 | 4308 | *trnK-*UUU*-rps16* | LSC |
|  | G | 12 | 4534 | *trnK-*UUU*-rps16* | LSC |
|  | C | 12 | 4586 | *trnK-*UUU*-rps16* | LSC |
|  | A | 11 | 8039 | *psbI-trnS-*GCU | LSC |
|  | T | 10 | 12297 | *atpF* intron | LSC |
|  | T | 11 | 12978 | *atpF-atpH* | LSC |
|  | T | 10 | 22588 | *rpoC1* intron | LSC |
|  | T | 11 | 30388 | *psbM-trnD-*UGC | LSC |
|  | T | 12 | 31702 | *trnT-*GGU*-psbD* | LSC |
|  | A | 11 | 46294 | *rps4-trnL-*UUA | LSC |
|  | T | 10 | 54616 | *atpB* | LSC |
|  | T | 14 | 55035 | *atpB-rbcL* | LSC |
|  | A | 15 | 59761 | *accD-psaI* | LSC |
|  | T | 11 | 66410 | *petL-petG* | LSC |
|  | A | 13 | 68486 | *rps18-rpl20* | LSC |
|  | T | 10 | 69491 | *rpl20-rps12* | LSC |
|  | A | 10 | 70717 | *clpP* intron | LSC |
|  | T | 10 | 78018 | *rpoA* | LSC |
|  | T | 15 | 83882 | *rps19-rpl2* | IR |
|  | G | 12 | 103309 | *trnA-*UGC intron | IR |
|  | T | 10 | 111705 | *ndhF-rpl32* | SSC |
|  | A | 10 | 112302 | *rpl32-trnL-*UAG | SSC |
|  | A | 15 | 113078 | *ccsA* | SSC |
|  | T | 10 | 119083 | *ndhA* intron | SSC |
|  | T | 11 | 124433 | *ycf1* | SSC |
|  | T | 11 | 124715 | *ycf1* | SSC |
|  | T | 10 | 125036 | *ycf1* | SSC |
|  | C | 12 | 132880 | *trnA-*UGC intron | IR |
|  | T | 15 | 152303 | *rpl2-rps19* | IR |
|  | TA | 10 | 8505 | *trnS-*GCU*-trnG-*UCC | LSC |
|  | AT | 10 | 19794 | *rpoC2* | LSC |
|  | AT | 10 | 45437 | *trnS-*GGA*-rps4* | LSC |
|  | TA | 10 | 46421 | *rps4-trnL-*UUA | LSC |
|  | TTC | 12 | 35310 | *psbC* | LSC |
|  | ATA | 12 | 64141 | *petA-psbJ* | LSC |
|  | AAT | 12 | 27530 | *rpoB-trnC-*GCA | LSC |
|  | TAGA | 12 | 204 | *trnH-*GUG*-psbA* | LSC |
|  | ATAA | 12 | 15427 | *atpI-rps2* | LSC |
|  | AATT | 12 | 36744 | *trnG-*GCC*-trnfM* | LSC |
|  | CAAA | 12 | 64534 | *petA-psbJ* | LSC |
|  | TAAA | 12 | 67216 | *trnP-*UGG*-psaJ* | LSC |
|  | TTTC | 12 | 71369 | *clpP* intron | LSC |
|  | AATA | 12 | 114258 | *ndhD* | SSC |
|  | AATT | 12 | 123911 | *ycf1* | SSC |

LSC: large single copy; SSC: small single copy; IR: inverted repeat

Table S2 Nucleotide diversity (Pi) values of the regions in *Echinacanthus*.

| Region | Pi | Region | Pi |
| --- | --- | --- | --- |
| *trnH-*GUG | 0.00676 | *trnH-*GUG*-psbA* | 0.06177 |
| *psbA* | 0.00898 | *psbA-trnK-*UUU | 0.02465 |
| *trnK-*UUU | 0.02643 | *trnK-*UUU*-rps16* | 0.04707 |
| *matK* | 0.02829 | *rps16-trnQ-*UUG | 0.04485 |
| *rps16* | 0.02504 | *trnQ-*UUG*-psbK* | 0.03812 |
| *trnQ-*UUG | 0.00694 | *psbK-psbI* | 0.06341 |
| *psbK* | 0.01703 | *psbI-trnS-*GCU | 0.0372 |
| *psbI* | 0.01315 | *trnS-*GCU*-trnG-*UCC | 0.04481 |
| *trnS-*GCU | 0 | *trnG-*UCC*-trnR-*UCU | 0.05 |
| *trnG-*UCC | 0.02718 | *trnR-*UCU*-atpA* | 0.033 |
| *trnR-*UCU | 0.00667 | *atpA-atpF* | 0.00862 |
| *atpA* | 0.01214 | *atpF-atpH* | 0.01842 |
| *atpF* | 0.01969 | *atpH-atpI* | 0.02164 |
| *atpH* | 0.00407 | *atpI-rps2* | 0.03147 |
| *atpI* | 0.01451 | *rps2-rpoC2* | 0.06849 |
| *rps2* | 0.01195 | *rpoC2-rpoC1* | 0.0184 |
| *rpoC2* | 0.01868 | *rpoC1-rpoB* | 0.02474 |
| *rpoC1* | 0.02182 | *rpoB-trnC-*GCA | 0.03613 |
| *rpoB* | 0.01552 | *trnC-*GCA*-petN* | 0.04474 |
| *trnC-*GCA | 0 | *petN-psbM* | 0.03702 |
| *petN* | 0.01961 | *psbM-trnD-*GUC | 0.02574 |
| *psbM* | 0.0101 | *trnD-*GUC*-trnY-*GUA | 0.05556 |
| *trnD-*GUC | 0 | *trnY-*GUA*-trnE-*UUC | 0.03279 |
| *trnY-*GUA | 0 | *trnE-*UUC*-trnT-*GGU | 0.03741 |
| *trnE-*UUC | 0 | *trnT-*GGU*-psbD* | 0.02467 |
| *trnT-*GGU | 0.00694 | *psbC-trnS-*UGA | 0.03882 |
| *psbD* | 0.00665 | *trnS-*UGA*-psbZ* | 0.02229 |
| *psbC* | 0.00738 | *psbZ-trnG-*GCC | 0.02229 |
| *trnS-*UGA | 0.00538 | *trnG-*GCC*-trnfM-*CAU | 0.03822 |
| *psbZ* | 0.01058 | *trnfM-*CAU*-rps14* | 0.01493 |
| *trnG-*GCC | 0 | *rps14-psaB* | 0.0336 |
| *trnfM-*CAU | 0 | *psaB-psaA* | 0.01852 |
| *rps14* | 0.0099 | *psaA-ycf3* | 0.02899 |
| *psaB* | 0.00922 | *ycf3-trnS-*GGA | 0.04188 |
| *psaA* | 0.01013 | *trnS-*GGA*-rps4* | 0.05426 |
| *ycf3* | 0.01168 | *rps4-trnL-*UUA | 0.03796 |
| *trnS-*GGA | 0.04023 | *trnL-*UUA*-trnF-*GAA | 0.03509 |
| *rps4* | 0.01045 | *trnF-*GAA*-ndhJ* | 0.03223 |
| *trnL-*UUA | 0.0098 | *ndhJ-ndhK* | 0.03462 |
| *trnF-*GAA | 0.00685 | *ndhK-ndhC* | 0.00909 |
| *ndhJ* | 0.01852 | *ndhC-trnV-*UAC | 0.03987 |
| *ndhK* | 0.00564 | *trnV-*UAC*-trnM-*CAU | 0.02591 |
| *ndhC* | 0.01377 | *trnM-*CAU*-atpE* | 0.03365 |
| *trnV-*UAC | 0.01133 | *atpB-rbcL* | 0.0252 |
| *trnM-*CAU | 0.00685 | *rbcL-accD* | 0.03603 |
| *atpE* | 0.01119 | *accD-psaI* | 0.03657 |
| *atpB* | 0.00924 | *psaI-ycf4* | 0.02469 |
| *rbcL* | 0.01081 | *ycf4-cemA* | 0.03349 |
| *accD* | 0.01402 | *cemA-petA* | 0.02167 |
| *psaI* | 0.00901 | *petA-psbJ* | 0.02685 |
| *ycf4* | 0.02106 | *psbJ-psbL* | 0.01235 |
| *cemA* | 0.01449 | *psbL-psbF* | 0 |
| *petA* | 0.01402 | *psbF-psbE* | 0 |
| *psbJ* | 0 | *psbE-petL* | 0.02214 |
| *psbL* | 0.00855 | *petL-petG* | 0.02462 |
| *psbF* | 0.00417 | *petG-trnW-*CCA | 0.04433 |
| *psbE* | 0.00397 | *trnW-*CCA*-trnP-*UGG | 0.03585 |
| *petL* | 0.02604 | *trnP-*UGG*-psaJ* | 0.01908 |
| *petG* | 0.00877 | *psaJ-rpl33* | 0.03455 |
| *trnW-*CCA | 0 | *rpl33-rps18* | 0.02198 |
| *trnP-*UGG | 0 | *rps18-rpl20* | 0.02146 |
| *psaJ* | 0 | *clpP-psbB* | 0.0348 |
| *rpl33* | 0.02736 | *psbB-psbT* | 0.03333 |
| *rps18* | 0.01797 | *psbT-psbN* | 0.03778 |
| *rpl20* | 0.01895 | *psbN-psbH* | 0.01402 |
| *clpP* | 0.01889 | *psbH-petB* | 0.03968 |
| *psbB* | 0.00808 | *petB-petD* | 0.02296 |
| *psbT* | 0.01961 | *petD-rpoA* | 0.02778 |
| *psbN* | 0.01263 | *rpoA-rps11* | 0.02113 |
| *psbH* | 0.01126 | *rps11-rpl36* | 0.00556 |
| *petB* | 0.01619 | *rpl36-infA* | 0.02577 |
| *petD* | 0.01744 | *infA-rps8* | 0.02033 |
| *rpoA* | 0.01677 | *rps8-rpl14* | 0.04571 |
| *rps11* | 0.01119 | *rpl14-rpl16* | 0.03175 |
| *rpl36* | 0.01316 | *rpl16-rps3* | 0.03717 |
| *infA* | 0.01068 | *rpl22-rps19* | 0.0102 |
| *rps8* | 0.00988 | *rps19-rpl2* | 0.02344 |
| *rpl14* | 0.014 | *rpl2-rpl23* | 0 |
| *rpl16* | 0.0215 | *rpl23-trnH-*CAU | 0.02516 |
| *rps3* | 0.01609 | *trnH-*CAU*-ycf2* | 0.01667 |
| *rpl22* | 0.02066 | *ycf2-ycf15* | 0.00653 |
| *rps19* | 0.01971 | *ycf15-trnL-*CAA | 0.01016 |
| *rpl2* | 0.01263 | *trnL-*CAA*-ndhB* | 0.00667 |
| *rpl23* | 0.02128 | *ndhB-rps7* | 0 |
| *trnH-*CAU | 0 | *rps7-trnV-*UAC | 0.00809 |
| *ycf2* | 0.01084 | *trnV-*GAC*-rrn16* | 0.00218 |
| *ycf15* | 0.00758 | *rrn16-trnI-*GAU | 0.00171 |
| *trnL-*CAA | 0 | *trnI-*GAU*-trnA-*UGC | 0.00192 |
| *ndhB* | 0.00211 | *trnA-*UGC*-rrn23* | 0.02083 |
| *rps7* | 0.00214 | *rrn23-rrn4.5* | 0 |
| *trnV-*GAC | 0 | *rrn4.5-rrn5* | 0.01008 |
| *rrn16* | 0.10479 | *rrn5-trnR-*ACG | 0.00213 |
| *trnI-*GAU | 0 | *trnR-*ACG*-trnN-*GUU | 0.00962 |
| *trnA-*UGC | 0.00485 | *trnN-*GUU*-ndhF* | 0.00919 |
| *rrn23* | 0.00178 | *ndhF-rpl32* | 0.05887 |
| *rrn4.5* | 0.00485 | *rpl32-trnL-*UAG | 0.06978 |
| *rrn5* | 0.00413 | *trnL-*UAG*-ccsA* | 0.07471 |
| *trnR-*ACG | 0 | *ccsA-ndhD* | 0.05556 |
| *trnN-*GUU | 0 | *ndhD-psaC* | 0.03927 |
| *ndhF* | 0.03634 | *psaC-ndhE* | 0.04398 |
| *rpl32* | 0.01283 | *ndhE-ndhG* | 0.04938 |
| *trnL-*UAG | 0.00625 | *ndhG-ndhI* | 0.0303 |
| *ccsA* | 0.03155 | *ndhI-ndhA* | 0.02469 |
| *ndhD* | 0.01901 | *ndhA-ndhH* | 0 |
| *psaC* | 0.01829 | *ndhH-rps15* | 0.0202 |
| *ndhE* | 0.01634 | *rps15-ycf1* | 0.04156 |
| *ndhG* | 0.01447 | *ndhH* | 0.01947 |
| *ndhI* | 0.01373 | *rps15* | 0.01648 |
| *ndhA* | 0.02363 | *ycf1* | 0.04399 |

Table S3 [Parameter](http://www.so.com/link?url=http://dict.youdao.com/search?q=Parameter&keyfrom=hao360&q=%E5%8F%82%E6%95%B0+%E8%8B%B1%E8%AF%AD&ts=1527064261&t=8b03d14344d9a77fb6cde77e04cbd71)s of Genes in branch model using Paml program

| Gene | lnL0 (Model0) | lnL1 (Model2) | df | P | ω= dN/dS (background clade/foreground clade) |
| --- | --- | --- | --- | --- | --- |
| *ndhC* | -635.754551 | -632.585682 | 1 | 0.0750544 | 0.15356/999.00000 |
| *ndhJ* | -913.466809 | -911.856615 | 1 | 0.2044645 | 0.15861/999.00000 |
| *psbK* | -326.392997 | -325.770681 | 1 | 0.4301879 | 0.32198/610.74036 |
| *psbN* | -230.238636 | -230.238626 | 1 | 0.9974769 | 0.10215/138.37585 |
| *rpl4* | -688.742987 | -688.742904 | 1 | 0.9927310 | 0.14207/463.65818 |
| *rpl16* | -2275.978589 | -2275.145204 | 1 | 0.3612955 | 0.38121/1.01789 |
| *rps4* | -1102.499900 | -1101.743822 | 1 | 0.3845587 | 0.33166/999.00000 |
| *rps15* | -549.226014 | -546.652447 | 1 | 0.1086625 | 0.26685/999.00000 |
| *rps18* | -565.351093 | -563.298265 | 1 | 0.1519238 | 0.14148/999.00000 |
| *rps19* | -588.393400 | -587.562837 | 1 | 0.3621098 | 0.16293/5.09877 |
| *infA* | -397.652972 | -395.534637 | 1 | 0.1455447 | 0.03663/999.00000 |
| *rpoB* | -1371.115877 | -1369.388220 | 1 | 0.1887104 | 0.31030/572.71798 |
